# Cyber-Downtime and Nursing Practice: Implications of Europe’s Network and Information Security (NIS2) Directive for Specialist Nursing Education

**DOI:** 10.7759/cureus.111183

**Published:** 2026-06-19

**Authors:** Giuseppe Fumai, Verdiana La Grotta

**Affiliations:** 1 Orthopedic and Neurological Rehabilitation, Rehcura Rehabilitation Facility, Adelfia, ITA; 2 Information, Communication, University Liaison, and Training, Azienda Sanitaria Locale Barletta-Andria-Trani (ASL BT), Barletta, ITA

**Keywords:** clinical resilience, cyber-downtime, cybersecurity in healthcare, nis2 directive, nursing competencies, nursing education, patient safety, specialist nursing

## Abstract

Healthcare now depends on digital systems for documentation, medication management, and continuity of care. When those systems fail during a cyber incident, nurses become the last reliable safeguard for patient safety at the bedside. Yet current training still leaves a gap between basic cyber awareness and the practical skills needed to manage care during downtime. The Network and Information Security (NIS2) Directive makes cybersecurity training a legal obligation for healthcare organizations, but nursing education across Europe has not yet turned that obligation into a formal clinical competency. Using Italy’s 2026 specialist nursing reform as a policy example, this editorial argues that cyber-downtime management, emergency paper documentation, incident communication, and simulation-based response should be embedded in specialist nursing curricula. Doing so would strengthen workforce readiness, improve patient safety, and reduce organizational vulnerability when digital systems fail. This editorial advances an evidence-informed normative proposal rather than an empirical study, and the proposed educational framework is preliminary and will require future validation.

## Editorial

Cyberattacks on healthcare organizations are no longer exceptional events: hospitals now face a specific and evolving cybersecurity threat landscape whose consequences extend far beyond data confidentiality into the operational continuity of clinical care. A sudden failure of hospital information systems can leave a ward without access to electronic records, medication traceability, or diagnostic reporting within minutes; when triggered by a cyberattack, this scenario escalates directly into a patient safety event, with sharply elevated risks of identification errors, medication administration failures, and documentation loss [1].

The European Union Agency for Cybersecurity (ENISA) reports that a significant proportion of cyberattacks targeting the health sector result in measurable disruption of care delivery, including diagnostic delays, therapeutic errors, and loss of clinical traceability [2]. The nurse is the healthcare professional present at the patient's bedside around the clock. During a downtime event, it is the nurse who must ensure safe medication administration, correct patient identification, and retrospective documentation of care activities performed without IT system support, tasks that require specific competencies that existing curricula largely do not address.

Dorosti et al. (2026) provide empirical evidence supporting this gap: while most nurses possess basic cyber awareness, a critical disconnect persists between theoretical knowledge and the practical skills required to manage care delivery under digital disruption, with 40% of ICU nurses in simulation unable to recognize an active cyberattack [3]. This editorial builds on that evidence to argue that the solution lies not in awareness campaigns but in structural educational reform grounded in EU law, a step the literature has not yet taken.

The Network and Information Security (NIS2) Directive (EU 2022/2555), transposed into national law across Member States from October 2024 onwards, establishes a binding cybersecurity framework for essential entities, a category that expressly includes public and accredited private healthcare organizations [4]. Article 21(2)(g) of the Directive requires essential entities to implement measures covering 'basic cyber hygiene practices and cybersecurity training.' This is a European statutory obligation, not a managerial discretion, whose non-fulfillment exposes organizations to administrative penalties and, under applicable national patient safety law, to potential organizational civil liability.

This regulatory imperative intersects with patient safety accountability frameworks across EU Member States, which consistently qualify care safety as a fundamental component of the right to health and require healthcare organizations to adopt adequate organizational structures to prevent its compromise. Despite this convergence of legal obligations, nursing cybersecurity training currently delivered in European healthcare organizations remains predominantly generalist, focusing on phishing recognition and credential management, content drawn from extra-clinical models, and lacking the operational dimension that defines cyber risk in clinical environments [3]. As Pelletreau et al. emphasize, training materials must be adapted to clinical workflows, modeled on proven patient safety interventions, and practiced regularly with all care teams; this level of clinical integration is absent from most existing nursing curricula [1].

A structural inconsistency drives this gap. Nursing professional frameworks across EU Member States were largely constructed in a pre-digital era and do not expressly recognize cyber-clinical competencies as a constitutive component of nursing practice. The result is a regulatory asymmetry: healthcare organizations are legally bound under NIS2 to train their nursing workforce, yet the professional frameworks that define nursing accountability do not structurally include these competencies. This ambiguity discourages systematic investment in cyber-clinical training and leaves frontline nurses exposed in scenarios that are now statistically predictable.

Italy's 2026 reform of specialist nursing education illustrates a concrete policy opportunity for EU Member States currently revising their nursing degree architectures. The Italian Ministry of University and Research established three new specialist nursing profiles: Primary Care and Community Nurse Specialist, Neonatal and Pediatric Care Nurse Specialist, and Intensive Care and Emergency Nurse Specialist, explicitly designed to develop advanced competencies aligned with European professional standards [5].

All three profiles operate in high-technology-dependency environments: community care relies on telemedicine and shared digital records; neonatal and pediatric settings manage connected monitoring devices and multidisciplinary data flows; intensive care and emergency settings depend on integrated systems whose unavailability has immediate prognostic consequences. Producing specialist nurses without cyber-clinical competencies would graduate professionals who are formally qualified but substantively unprepared for a risk category that ENISA identifies as structurally recurrent across EU health systems [2]. The Italian case should be read as an illustrative policy example rather than a uniquely national exception; comparable nursing education reforms are underway across multiple EU Member States.

The recommendations advanced here derive from a focused analysis of the applicable European and national regulatory framework (NIS2 Directive [4], the 2026 Italian specialist-nursing decree [5], the General Data Protection Regulation (GDPR), and national patient-safety legislation) combined with a narrative reading of the emerging empirical literature on nurses’ cyber-preparedness [1,3]; they represent a policy- and evidence-informed proposal rather than the product of primary data collection or formal expert consensus. Drawing on the operational preparedness framework proposed by Dorosti et al. [3] and the clinical continuity evidence synthesized by Pelletreau et al. [1], this editorial proposes that academic regulations governing new specialist nursing programs across EU Member States incorporate a dedicated cyber-clinical competency module within core curricula. The essential content of such a module should include: the applicable European and national regulatory framework (NIS2 Directive, GDPR, and relevant patient safety legislation); clinical downtime management protocols, including analog continuity-of-care procedures and emergency paper-based documentation; incident notification procedures and information flows between clinical management, data protection officers, and national cybersecurity authorities; and simulation-based downtime exercises specific to each specialist clinical setting. Effective preparedness further requires that these competencies be developed through interdisciplinary co-design and joint exercises with hospital information technology and information security teams, with the active engagement of clinical leadership, rather than in isolation from the technical functions on which downtime response depends.

The following framework outlines how cyber-clinical content could be structured across the nursing education continuum, distinguishing the foundational competencies appropriate to generalist (Bachelor’s) preparation, data-protection fundamentals, phishing recognition, and secure credential management from the advanced, setting-specific competencies required at specialist (Master’s) and postgraduate levels, namely clinical downtime management, analog continuity-of-care protocols, incident notification, and simulation-based downtime exercises (Table 1).

**Table 1:** Proposed integration of cyber-clinical competencies across nursing education levels. Authors' own elaboration. These cyber-clinical competencies are not included in the curricular framework established by Italian Ministerial Decree of 6 February 2026, no. 159 [5]. GDPR: General Data Protection Regulation; NIS2: Network and Information Security

Educational level	Proposed cyber-clinical content
Bachelor's Degree (Generalist Nursing)	GDPR and health data protection fundamentals; phishing and social engineering recognition; secure credential management; basic clinical cybersecurity principles
Specialist Master's Degree	Clinical downtime management; analog continuity-of-care protocols without IT systems; NIS2 obligations and incident notification; multiprofessional clinical data governance; simulation-based downtime exercises
University Postgraduate Programs	Advanced healthcare cybersecurity; digital continuity nursing leadership; integration of IT security and care safety frameworks; clinical-organizational evaluation of health information systems
Continuing Professional Development (CPD/CME)	Periodic regulatory updates; operational downtime simulations; post-incident clinical debriefing; NIS2-mandated organizational training with recognized CPD credit

Integrating cyber-clinical training into academic curricula is the strategic long-term measure, but it does not address the immediate vulnerability of the currently employed nursing workforce, trained before the mass digitalization of hospitals, on whom NIS2 obligations fall most directly. For this group, dedicated continuing professional development pathways with recognized mandatory hours under applicable NIS2 transposition law are an essential parallel intervention. National nursing professional bodies could contribute by issuing guidance documents to define minimum competency standards and orient training provision.

Translating this proposal into practice will encounter non-trivial implementation barriers that merit explicit acknowledgement. These include faculty preparedness, as few nurse educators currently combine clinical expertise with cybersecurity competence; resource requirements for simulation infrastructure and scenario development; curriculum overload within already dense specialist programs; and substantial heterogeneity across European healthcare systems in digital maturity, NIS2 transposition, and educational governance. Phased curricular integration, shared simulation resources, and continuing professional development pathways for the existing workforce represent pragmatic mitigations, while a dedicated feasibility and cost analysis remains a necessary component of the implementation-research agenda.

This editorial advances an evidence-informed normative proposal, not an empirically validated intervention. The curriculum framework in Table 1 has not yet been tested for feasibility, uptake, or educational outcomes in any EU jurisdiction. Future research should evaluate the implementation of cyber-clinical modules within specialist nursing programs using validated outcome measures and assess the impact of structured downtime simulation on nurse preparedness and patient safety indicators. No effectiveness data yet support the specific educational interventions recommended here, and this is the principal limitation of the present contribution. Although controlled evidence on the educational effectiveness of simulation-based cyber-downtime training is not yet available, the modality builds on an established patient-safety practice, regular, team-based downtime drills [1], and simulation has already proved able to expose the preparedness gap, with a substantial proportion of ICU nurses failing to recognize an active cyberattack under simulated conditions [3]. Generating outcome evidence for such training is accordingly a priority for the research agenda.

The digitalization of European healthcare is irreversible. Cybersecurity is not peripheral to nursing: it is a constitutive dimension of care safety, and the nurse stands at the intersection of clinical and digital risk. The available evidence, though still limited, points to a preparedness gap across several countries and care settings [1-3]. The NIS2 Directive has already established the legal framework for action. What is currently missing is its translation into nursing education.

The ongoing window of specialist nursing education reform across EU Member States offers a concrete opportunity to close this gap structurally. Realizing it requires coordinated engagement by universities, national nursing professional bodies, ministries of health and education, and national cybersecurity authorities. The cost of inaction is measurable: it is counted in adverse events, institutional liability, and the professional exposure of nurses managing clinical crises with competencies the system has not equipped them to deploy.

The key message is that cyber-clinical competencies should become a formal component of specialist nursing education because cybersecurity is now a legal, operational, and patient-safety necessity.
